# A tandem CD19/CD20 CAR lentiviral vector drives on-target and off-target antigen modulation in leukemia cell lines

**DOI:** 10.1186/s40425-017-0246-1

**Published:** 2017-05-16

**Authors:** Dina Schneider, Ying Xiong, Darong Wu, Volker Nӧlle, Sarah Schmitz, Waleed Haso, Andrew Kaiser, Boro Dropulic, Rimas J. Orentas

**Affiliations:** 1 Lentigen Technology, Inc., 910 Clopper Rd., Gaithersburg, MD 20878 USA; 2 Miltenyi Biotec GmbH, Bergisch Gladbach, Germany

**Keywords:** CAR T, Tandem -targeting CAR, Lentiviral vector, Adoptive immunotherapy, Hematologic malignancy, Tumor antigen escape, CD19, CD20, CD22

## Abstract

**Background:**

Clinical success with chimeric antigen receptor (CAR)- based immunotherapy for leukemia has been accompanied by the associated finding that antigen-escape variants of the disease are responsible for relapse. To target hematologic malignancies with a chimeric antigen receptor (CAR) that targets two antigens with a single vector, and thus potentially lessen the chance of leukemic escape mutations, a tandem-CAR approach was investigated.

**Methods:**

Antigen binding domains from the FMC63 (anti-CD19) and Leu16 (anti-CD20) antibodies were linked in differing configurations to transmembrane and T cell signaling domains to create tandem-CARs. Expression on the surface of primary human T cells was induced by transduction with a single lentiviral vector (LV) encoding the tandem-CAR. Tandem-CARs were compared to single antigen targeting CARs in vitro and in vivo, and to an admixture of transduced cells expressing each CAR in vivo in immunodeficient (NSG) disease-bearing mice.

**Results:**

Tandem constructs efficient killed the Raji leukemia cell line both in vitro and in vivo. Tandem CARs generated less cytokine than the CD20 CAR, but similar to CD19 CARs, on their own. In co-culture experiments at low effector to target ratios with both single- and tandem- CAR-T cells, a rapid down-modulation of full-length CD19 expression was seen on leukemia targets. There also was a partial down-modulation of CD22, and to a lesser degree, of CD20. Our data also highlight the extreme sensitivity of the NALM-6 cell line to general lymphocyte-mediated cytotoxicity. While single and tandem constructs were effective in vivo in a standard setting, in a high-disease burden setting, the tandem CAR proved both effective and less toxic than an admixture of transduced T cell populations expressing single CARs.

**Conclusion:**

Tandem CARs are equally effective in standard disease models to single antigen specificity CARs, and may be both more effective and less toxic in a higher disease burden setting. This may be due to optimized cell killing with more moderate cytokine production. The rapid co-modulation of CD19, CD20, and CD22 may account for the ability to rapidly evolve escape mutants by selecting for leukemic clones that not require these target antigens for continued expansion.

**Electronic supplementary material:**

The online version of this article (doi:10.1186/s40425-017-0246-1) contains supplementary material, which is available to authorized users.

## Background

Adoptive immunotherapy for cancer with genetically engineered autologous human T cells is currently being evaluated in numerous centers. One common approach to creating a cell population for adoptive immunotherapy is to isolate T cells by apheresis from the patient and to transduce these cells ex vivo with retroviral or lentiviral vectors that integrate into the host genome and express a chimeric antigen receptor (CAR), reviewed in [1]. Chimeric antigen receptors are created by linking functional sequence domains from different subunits of immunologically active proteins. For example, an scFv domain created from the V_H_ and V_L_ domains of an anti-CD19 or anti-CD20 antibody can be linked to transmembrane sequences derived from CD28 or CD8, and then linked to the intracellular signaling domains derived from the CD3-zeta chain and CD28 or CD137 [2, 3]. The CAR thus confers both a binding domain derived from the scFv and the linked signaling domains in a single transmembrane protein that allows activation of a vector-transduced T cells. This transduced T cell population (CAR-T) can now functionally target cells bearing the cognate antigen for destruction by active cytolysis, and by indirect immune effector mechanisms marshaled by the production of cytokines, such as interferon-gamma (IFNγ), interleukin-2 (IL-2), and tumor necrosis factor-alpha (TNFα). Adoptive immunotherapy with chimeric antigen receptor modified T cells that specifically target CD19 has proven efficacy against pediatric pre-B ALL [4, 5]. The effectiveness of CAR-modified T cells in adult hematologic malignancies has been more heterogeneous.

While the experience with anti-CD19 CAR-T therapy at the University of Pennsylvania with 3 CLL patients seemed to indicate a universal positive response, the Surgery Branch at the NCI reported a mixture of partial responses, stable disease, and one complete response in a diverse collection of 8 patients with adult B cell malignancies [6, 7]. Thus, the anti-CD19 CAR is not universally effective and may benefit from further enhancement of its anti-tumor targeting potential. The laboratory of Thomas-Tikhonenko has elegantly described the escape mechanisms employed by B-ALL during anti-CD19 CAR-T therapy which include alternative splicing of CD19, frameshift mutations, and missense mutations [8]. One means to both broaden the target range of a CAR-T product as well as to target malignancies with greater effect is to include two binding domains in a single CAR structure. Tandem CD19- and CD20-expressing malignancies include chronic lymphocytic leukemia (CLL), hairy cell leukemia (HCL), mantle cell lymphoma (MCL), prolymphocytic leukemia (PLL), and splenic lymphoma with villous lymphocytes (SLVL) [9]. A single CAR vector targeting both antigens would target a broader variety of hematologic malignancies, and potentially target them more effectively. We modeled the increased effectiveness of a tandem-CAR construct by testing the expression of both target antigens on the CD19^+^CD20^+^ Raji cell line upon co-culture with a series of both single specificity and tandem-specific CAR-T constructs. We demonstrate here a rapid CD19 target antigen down modulation mechanism present in this leukemia cell line. Surprisingly, target antigen down-modulation also included non-targeted B cell receptor molecules, including CD22, which is not targeted by any of the vector constructs. The more rapid loss in target cell number with our tandem targeting vector indicates the presence of stronger anti-leukemic immune pressure. The kinetics of the escape from immune pressure by the leukemia cell line, especially when single specificity CARs are used, indicates that antigenic modulation is a pre-existing property of the leukemia cell, as opposed to a mechanisms of target antigen modulation or loss that is dependent on selection for genetic escape mutants.

## Methods

### Cell lines (PBMC and targets)

All cell lines and reagents were purchased from American Tissue Culture Collection (ATCC, Manassas, VA), unless otherwise noted. The Burkitt lymphoma cell line Raji the acute lymphocytic leukemia cell lines REH and NALM-6 (ACC-128 DSMZ, Leibniz Institute DSMZ, Braunschwieg, Germany), as well as the chronic myelogenous leukemia line K562 were cultured in RPMI-1640 medium supplemented with 10% heat-inactivated fetal bovine serum (FBS, Hyclone, Logan, UT) and 2 mM L-Glutamax (Thermo Fisher Scientific, Grand Island, NY). The human embryonic kidney cell line 293 T was propagated in Dulbecco’s modified Eagle medium supplemented with 10% heat-inactivated FBS.

Single-cell clones of luciferase-expressing cell lines were generated by stably transducing wild-type tumor lines with lentiviral vector encoding firefly luciferase (Lentigen Technology, Inc., Gaithersburg, MD), followed by cloning and selection of luciferase-positive clones. The mouse-adapted Raji-luc line was generated by engrafting a Raji clone stably expressing firefly luciferase into NSG mice (NOD.Cg-*Prkdc* ^*scid*^ *Il2rg* ^*tm1Wjl*^/SzJ), The Jackson Laboratory Sacramento, CA), isolating the engrafted Raji-luc tumor cells from mouse spleens by either positive (CD19 microBeads, human, Miltenyi Biotec, Bergisch Gladbach, Germany) or negative selection (mouse cell depletion kit, Miltenyi Biotec), expanding in culture, and re-cloning to facilitate the selection of clones with high expression of firefly luciferase, as previously described for the NALM6 cell line [10].

Whole blood was collected from healthy volunteers at Oklahoma Blood Institute (OBI) with donors’ written consent. Processed buffy coats were purchased from OBI (Oklahoma City, OK). The CD4-positive and CD8-positive human T cells were purified from buffy coats via positive selection using a 1:1 mixture of CD4- and CD8- MicroBeads (Miltenyi Biotec) according to manufacturer’s protocol.

### Creation of Chimeric Antigen Receptor (CAR) – expressing vectors

CAR antigen-binding domains, scFv, sequences were derived from the mouse hybridoma FMC-63 for CD19 (FMC-63: AA 1-267, GenBank ID: HM852952.1) and Leu-16 for CD20 [11], entire sequence of V_L_ and V_H_), as in *Additional files* section.

CAR19A, CAR19B and CAR20A were generated by linking scFv of each antibody in frame to CD8 hinge and transmembrane domains (AA 123-191, Ref sequence ID NP_001759.3), 4-1BB (CD137, AA 214-255, UniProt sequence ID Q07011) transactivation domain and CD3 zeta signaling domain (CD247, AA 52-163, Ref sequence ID: NP_000725.1.). Constructs 19A and 19B were identical, except for the flexible linker connecting the variable H and L chains of the scFv binding domain, employing the Whitlow linker in 19A [12] and a (GGGGS)_3_ linker in 19B. Tandem targeting constructs, CAR1920 and CAR2019, were generated in a similar manner. The scFv regions of 19A and 20A were linked in sequence by a flexible interchain linker (GGGGS)_5_, followed by CD8, 4-1BB and CD3 zeta domains. Leader sequence from human granulocyte macrophage colony stimulating factor receptor alpha subunit was included in all constructs, as described in [13]. CAR constructs sequences were codon optimized (DNA2.0, Newark, CA) and cloned into a third generation lentiviral plasmid backbone (Lentigen Technology Inc., Gaithersburg, MD) under the regulation of a human EF-1α promoter.Lentiviral vector (LV) containing supernatants were generated by transient transfection of HEK 293 T cells, as previously described [14]. Harvested pelleted lentiviral supernatants were stored at −80 °C.

### Primary T cell transduction

Selected CD4+ and CD8+ human primary T cells from normal donors were cultivated in TexMACS medium (serum-free) supplemented with 40 IU/ml IL-2 at a density of 0.3 to 2 × 10^6^ cells/ml, activated with CD3/CD28 MACS® GMP TransAct reagent (Miltenyi Biotec) and transduced on day 3 with lentiviral vectors encoding CAR constructs in the presence of 10 ug/ml protamine sulfate (Sigma-Aldrich, St. Louis, MO) overnight, and media exchanged on day 4. On day 5, cultures were transferred to TexMACS medium supplemented with 200 IU/ml IL-2, and propagated until harvest on day 10–13.

### Immune effector assays (CTL and cytokine)

To determine cell-mediated cytotoxicity (CTL assay), 5,000 target cells stably transduced with firefly luciferase were combined with CAR T cells at various effector to target ratios and incubated overnight. SteadyGlo reagent (Promega, Madison WI) was added to each well and the resulting luminescence was analyzed on an EnSpire plate reader (Perkin Elmer, Shelton, Connecticut) and recorded as counts per second (sample CPS). Target only wells (max CPS) and target only wells plus 1% Tween-20 (min CPS) were used to determine assay range. Percent specific lysis was calculated as: (1-(sample CPS-min CPS)/(max CPS-min CPS)). For cytokine release assays, effector and target cells were combined at ratio 10:1 and incubated overnight. Harvested supernatants were analyzed for secreted cytokines using MACSplex human 12 cytokine bead array kit (Miltenyi Biotec) as per manufacturer’s instructions. Strong induction of IFNγ, TNFα, IL-2 and GM-CSF were detected in CAR T-treated groups. The following cytokines could not be detected: IL-4, IL-5, IL-6, IL-12p70, IL-17A, IL-10, IFNα. IL-9 was detected in some samples at low levels and was not reported. All samples were in duplicate or triplicate. Unless otherwise noted, all data shown is representative of three or more independent experiments.

### Western blot

Two million CAR T cells were washed twice in cold PBS (Lonza, Walkersville, MD), then lysed in 100 ul cold RIPA buffer (Sigma-Aldrich, St. Louis, MO) containing a protease and phosphatase inhibitor cocktail (Thermo-Fisher Scientific, Grand Island, NY). The lysate was incubated at 4 °C for 20 min, pelleted at 13000 RPM in a table top centrifuge at 4 °C for 10 min, and supernatants collected and frozen at -20 °C. Samples were denatured at 70 °C in reducing loading buffer (Invitrogen) for 10 min and resolved on 4–12% gradient SDS-PAGE gel under reducing conditions in MOPS buffer (Thermo-Fisher Scientific, Grand Island, NY) according to manufacturer’s protocol. Proteins were transferred to 0.45 μm nitrocellulose transfer membrane (BioRad, Hercules, CA) and probed with antibody against pan-CD3 zeta (Clone ab40804, Abcam, Cambridge, MA). Bands were developed using Vectastain ABC-AMP reagent kit (Vector Laboratories, Burlingame, CA) according to manufacturer’s protocol and bands were visualized and quantified on an Odyssey imaging system with Image Studio lite software (LI-COR, Lincoln, Nebraska). Western Blot for CD19 was also performed on Raji tumor cells. Briefly, after overnight incubation of Raji cells with CAR T cells, CD3-positive cells were depleted via LD columns using CD3 magnetic beads (Miltenyi Biotec) according to manufacturer’s protocol, and the recovered Raji cells were processed as above. Specific bands were detected using antibodies directed to CD19 C-terminus (sc-69735, Santa Cruz, CA), and beta-actin (8457, Cell Signaling Technology, Danvers, MA). Band intensity was quantified by Image Studio software (LI-COR, Lincoln, Nebraska). Relative band intensity of full length CD19 and Δ exon 2 CD19 isoform was calculated as signal CD19/signal β actin.

### Flow cytometric analysis

All cell staining reagents for flow cytometry were from Miltenyi Biotec, unless otherwise noted. One million CAR T transduced cells were harvested from culture, washed two times in cold staining buffer (AutoMACS solution with 0.5% bovine serum albumin) and pelleted at 350 xg for 5 min at 4 °C.

CAR surface expression on transduced T cells was detected by staining with protein L-biotin conjugate (stock 1 mg/ml, 1:1000 dilution, GenScript, Piscataway, NJ) for 30 min at 4 °C, followed by two washes and staining with streptavidin-PE conjugate for 30 min at 4 °C (stock: 1.0 ml, 1:200 dilution, Jackson ImmunoResearch Laboratories, West Grove, PA). Non-transduced cells and transduced cells stained with streptavidin-PE only, were used as negative controls. Anti-CD4 antibody was employed to determine CD4 to CD8 ratio of CAR T positive population, and was added during the second incubation step. Dead cells were excluded by 7AAD staining (BD Biosciences, San Jose, CA). Cells were washed twice and resuspended in 200 ul Staining Buffer before quantitative analysis by flow cytometry.

Specific CAR T staining was carried out with Fc-tagged-CD19 peptide (described below, at 1 μg/ml) by incubating with cells for 15 min at 4 °C, followed by incubation with anti-Fc-AF647 F(ab’)_2_ fragment at 4 °C for 15 min (Jackson Immuno Research, 1:200) and detected in the APC channel. Biotinylated CD20 peptide (Bachem, Torrance, CA) and strepatvidin PE (both at 1 μg/ml) were added to cells simultaneously and incubated at room temperature for 10 min in the dark. Flow cytometric analysis was performed on a MACSQuant®10 Analyzer (Miltenyi Biotec). Characterization of target tumor lines and luciferase-positive sub clones was performed using CD19-FITC, CD20 VioBlue, and CD22-APC antibodies. Dead cells were excluded from analysis by 7AAD staining (BD Biosciences, San Jose, CA).

### Generation of Fc-tagged CD19 peptide

For production of recombinant human CD19 peptide, the extracellular domain (amino acids 20-291, Uniprot P15391) was fused to human IgG1 Fc (CD19-Fc) and expressed by transduction in HEK293 cells in a CMV-driven mammalian expression vector. Transfected cells were cultured in DMEM, 5% FBS, and cell culture supernatant containing CD19-Fc harvested after 10 and 20 days of incubation in HYPER*Flask*® cell culture vessels (Corning). After centrifugation to remove cell debris and 0.22 μm sterile filtration, CD19-Fc was purified by protein A chromatography (HiTrap MabSelect, GE Healthcare) and stored in PBS at 4 °C. Purity was > 97% as determined by SDS-PAGE and Coomassie Blue staining. Identity of CD19-Fc was confirmed by intact mass spectrometry and peptide mass fingerprint analyses after trypsin digestion(Miltenyi Biotec, Bergisch Gladbach, Germany).

### In vivo analysis of CAR-T activity

All animal studies were approved by Jackson Laboratory Animal Care and Use Committee (Sacramento, CA). A half million mouse-adapted Raji-luc cells were injected into the tail vein of NSG (NOD.Cg-*Prkdc* ^*scid*^ *Il2rg* ^*tm1Wjl*^/SzJ) mice. On day 6 following Raji-luc injection, tumor engraftment was measured by i.p. injection of 150 mg/kg luciferin and imaging 10 min later for 40 s on a Xenogen IVIS-200 instrument (Caliper Biosciences, now Perkin Elmer, Shelton, Connecticut). Images were analyzed using Living Image, version 4.1, software (Perkin Elmer) and the bioluminescent signal flux for each mouse was expressed as average radiance (photons per second per cm^2^ per steradian). CAR T cells were administered to mice via tail vein injection on Day 7. Imaging was performed on days 4, 6, 11, 14, 18, 25, 32, 46 following injection to establish the kinetics of tumor growth and eradication by CAR T cells.

For the high tumor burden in vivo study, NSG mice were injected *i.v.* with Raji-luciferase cells on Day 0. Mice were distributed equally to study groups on day 11 based on tumor burden. CAR T cell preparations of tandem-CAR 2019 or a combination two single CAR T preparations mixed at equal CART^+^ cell numbers (19A + 20A, 19B + 20A) were then administered *i.v.* on study day 12. All CAR T preparations were tested at 5 × 10^6^ total CAR T cells/mouse. Non-transduced T cells from the same donor (N.T.) and Tumor alone group served as controls. On study days 18 and 25, tumor growth was assessed based on mouse whole body average radiance. *N* = 6/group.

### In vitro analysis of leukemia immune-evasion

To analyze Raji tumor escape variants, CAR T and Raji cells were combined in vitro at an effector to target ratio of 1:1. For surviving Raji cells, surface expression of CD19, CD20 and C22 was determined by flow cytometry after overnight incubation and at day 4 of co-culture. Cultures were harvested, washed, and stained with antibodies specific for CD3-PE, CD19-FITC, CD20-VioBlue, CD22-APC (Miltenyi Biotec), and 7AAD. To facilitate the analysis of the surviving Raji cells in each Raji:T cell co-culture, we gated on the population of CD3-negative 7AAD-negative (i.e. live Raji) cells and this population was then analyzed for residual surface expression of CD19, CD20 and CD22.

In the transwell co-culture assay, 5 × 10^5^ each of CAR T and Raji cells were seeded in the bottom compartment of a 24- transwell plate (Costar, REF 3470, 0.4 μM pore membrane), in 1 ml of TexMACS medium. 2.5 × 10^5^ Raji cells were seeded in the upper transwell compartment in TexMACS medium in the absence of T cells. Following overnight incubation, cells from the upper and lower compartment were analyzed by flow cytometry as described above. Percentage expression of CD19, CD20 and CD22 on Raji cells for each group was measured. Data presented shows the average value + SD from three independent experiments, from three different donors. Statistical analysis was performed by one-way ANOVA followed by Dunnett’s multiple comparisons test vs Raji alone control, *p < 0.01.”

### Statistical analysis

Statistical analysis was performed using GraphPad Prism 7.01 software. In in vitro killing assays, group means for replicate determinations were compared by two-way analysis of variance (ANOVA) followed by Dunnett’s multiple comparisons test to identify differences between individual treatment groups and the non-transduced controls. In the first in vivo study, IVIS radiance data was analyzed on experimental day 25, the last measurement day when all groups had viable mice, by two-way ANOVA followed by Dunnett’s multiple comparisons test vs no treatment group. Analysis of CD19, CD20 and CD22 expression in Raji and NALM-6 cells after overnight and 4 days of co-culture with CAR T cells was performed by one-way ANOVA followed by Dunnett’s multiple comparisons test vs N.T. (non-transduced T cells from the same donor) control. Analysis of CD20/CD19 binding ratios in tandem-CARs was performed by student t-test.

## Results

**Fig. 1:**
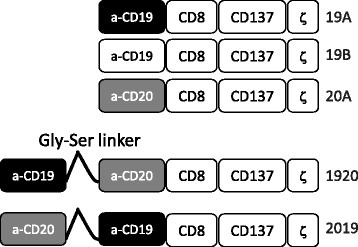
Construction of CARs targeting CD19 and CD20. Anti-CD19 and anti-CD20 single targeting CAR constructs were generated by linking single chain fragment variable region of monoclonal antibody FMC-63 (CD19) of Leu-16 (CD20) in frame to CD8 hinge and transmembrane domain, the 4-1BB (CD137) signaling domain and the CD3 zeta signaling domain. Constructs 19A and 19B differ only in the linker sequence connecting the heavy and the light chains of FMC63. Tandem targeting constructs 2019 and 1920 were generated in a similar manner to single targeting constructs, except that the single chain fragment variable regions of CD20 and CD19 were linked to each other sequentially by a flexible linker, followed by CD8, 4-1BB and CD3 zeta domains

To study the effectiveness of tandem CD19 and CD20 targeting CARs, sequences encoding the antigen binding domains from the murine antibodies FMC63 and Leu16 were linked with a (GGGGS)_5_ sequence. For all constructs in this study, the linking, transmembrane and signaling domains were identical, each encoding a human CD8-derived hinge and transmembrane domain, a CD137 signaling domain, and a CD3-zeta-derived signaling domain, as previously reported [13], Fig. 1. The heavy and light chains of FMC63 were linked together into an scFv structure as originally published (GenBank ID HM852952.1, AA 130-148, known as the Whitlow linker) [12] while the heavy and light chains of Leu16 were linked by a (GGGGS)_3_ sequence. Tandem-CARs were created by joining the FMC63-derived and Leu16-derived heavy and light chain sequences in a single transcript with a multiple-GGGGS sequence as well. Unlike their single specificity counter-parts, the tandem-CARs can induce activation of the CAR-expressing T cells by encountering target cells that express either CD19 or CD20.

**Fig. 2:**
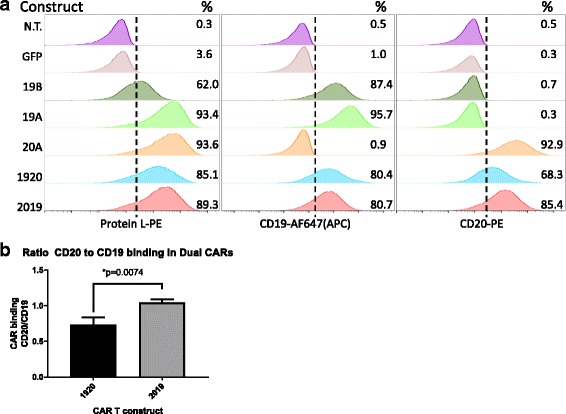
Surface expression of single and tandem-CAR T constructs on human primary T cells. **a** CAR T expression was determined by flow cytometry. T cells were activated with Transact CD3 CD28 reagent in the presence of IL-2, and transduced with LV as described in Methods. On culture day 10, viable transduced T cells (7-AAD negative) were assayed for CAR surface expression using one of three staining reagents: Protein L (column 1), CD19 Fc followed by anti-Fc-AF647 (column 2), or CD20-biotin followed by streptavidin-PE staining (column 3). The LV used in transduction is listed to the *left* of each row. Percentage of CAR T-positive populations in relation to non-transduced T cell control is noted in the right-hand corner of each histogram. GFP-transduced cells served as an additional negative control. Representative data of three separate donors is shown. **b** The ratio of CD19 and CD20 antigen binding by each tandem-CAR is expressed as the ratio of percent cells bound by CD20 biotin vs CD19 Fc. The average + SD of three separate experiments using three donors is shown, **p < 0.01

Primary human T cells were activated with anti-CD3/CD28 nanomatrix in 40 IU/ml IL-2 and transduced with lentiviral vectors encoding CARs 3 days later, followed by expansion in 200 IU/ml IL-2, which was maintained throughout the culture period. CAR expression was measured on the surface of transduced T cells by flow cytometry using biotinylated protein L, followed by staining with a Streptavidin-PE, Fig. 2a. Expression of CARs on the transduced T cell surface ranged from 61 to 93%. To verify that both scFv binding domains were intact we also co-incubated CAR T cells with a CD19-Fc fusion protein or with biotinylated CD20-peptide (see Methods). In tandem-specific constructs, both domains retained the ability to bind target antigen. For CAR2019, protein L staining gave 89% expression and CD19-Fc expression was 80%, and CD20-peptide expression was 85%. For CAR 1920, protein L staining gave 85% CAR expression, CD19-Fc staining yielded 80%, and CD20 peptide 68%. This observation was reproduced in CAR T cells generated from three separate donors.

To compare differences in antigen binding we calculated the CAR T CD20/CD19 binding ratio. This ratio was defined as the percentage of the tandem-CAR T cells positively stained with the CD20 soluble peptide, divided by the percentage of CAR T cells positively stained by the CD19-Fc peptide in the same sample. The mean CD20/CD19 binding ratio for three separate donor T cell transductions was 0.74 ± 0.06 for CAR1920, and 1.05 ± 0.02 for CAR 2019 (Fig. 2b). Thus, whereas in CAR T construct 2019 the binding of the soluble CD19 and CD20 peptides is similar and comparable to the Protein L binding, the CD20 binding in the CAR 1920 construct may be partially sterically hindered.

**Fig. 3:**
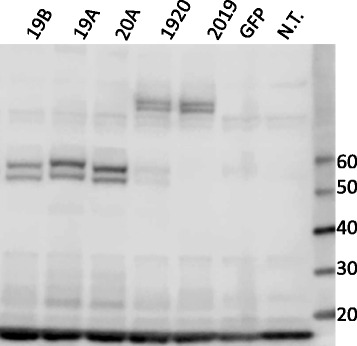
Western blot of CAR T proteins in primary human T cells. Cell lysates prepared from human primary T cells transduced with single (19B, 19A, 20A) or tandem-CAR19 and CAR20 (1920, 2019) constructs were resolved on a gradient (4–12%) SDS-PAGE gel and probed with an antibody against CD3-zeta. T cells from the same donor transduced with a lentiviral vector encoding *green* fluorescent protein (GFP), or non-transduced T cells (N.T.) served as controls. Molecular weight markers, in KDa, are listed to the *right*

To verify that the flow profiles were due to a single, larger, transcript, molecular weights of the CAR protein were verified by Western blot under reducing conditions, Fig. 3. Specific bands of the expected size were detected for the single (54 KDa)- and tandem (81 KDa) CARs. To ascertain relative expression levels, band intensities were compared by calculating the ratio of CAR associated zeta chain signal to endogenous CD3 zeta. Normalizing the CAR expression levels in the CAR 19B single chain vector to 1 (arbitrary unit), the expression for 19A, 20A, 1920, and 2019 were 1.8, 1.5, 0.9 and 1.2, respectively. Thus, the expression levels of tandem-CARs fall within the variability seen with single CAR expression vectors. Given the ability to create LV that express tandem-CARs, and now having demonstrated the expression of full-length CAR proteins on the T cell surface that express two functional scFv, we next evaluated the anti-leukemic activity of tandem-CAR constructs in in vitro assays.

Human primary T cells were transduced with LV encoding CAR constructs (19A, 19B, 20A, 1920, 2019, see Methods), then incubated for 18 h with the Raji, NALM-6, REH, K562 or 293 T cell lines, stably transduced with firefly luciferase, for luminescence based in vitro killing assays. All leukemia lines tested express CD19 on their surface, while the negative controls, K562 and 293 T do not. CD20 expression varied between tumor lines. The Raji line is CD20 positive, while REH are CD20 negative, as are the control lines K562 and 293 T. NALM-6 line has a weak but detectable expression of CD20. As additional controls, K562 lines were created that express CD19 (K562-19^+^), or CD20 (K562-20^+^).

**Fig. 4:**
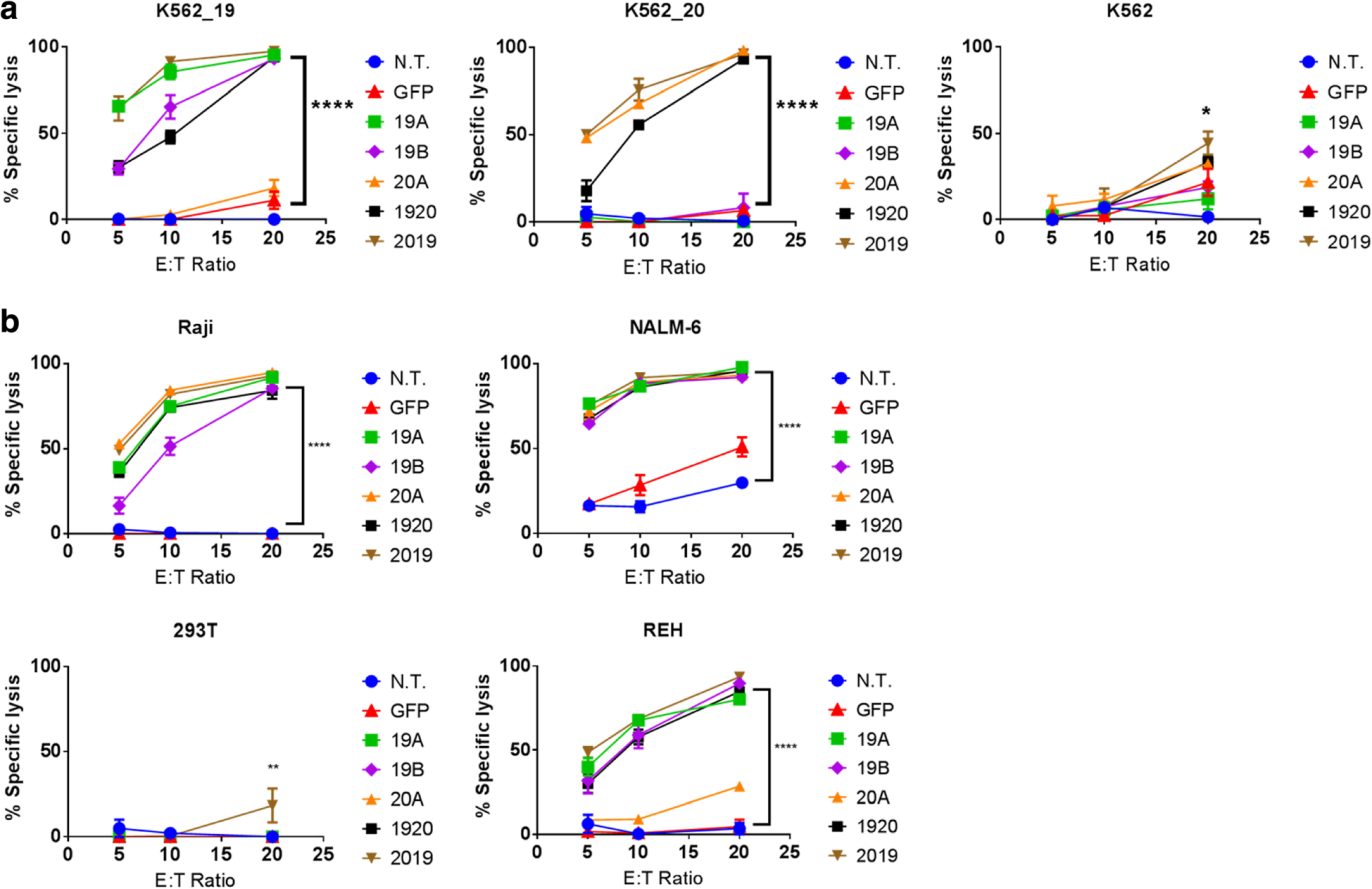
CAR T cytotoxicity in vitro*.* Luciferase-based cytotoxicity assays were performed using, **a**) K562, K562 CD19^+^, or K562 CD20^+^ cell lines, or **b**) leukemia or lymphoma cell lines (Raji, NALM6, REH), stably transduced with luciferase. CAR T cells and target tumor cells were co-incubated overnight at the listed effector to target (E:T) ratios, x-axis. Differences between groups were determined using 1-way ANOVA followed by Dunnett’s post-hoc test. Mean + SD, ****p < 0.0001, **p < 0.01 vs non-transduced control from the same donor (N.T.)

K562-19^+^ were lysed by the CAR 19A and 19B constructs, tandem-CAR constructs 1920 and 2019, but not the single 20A CAR, Fig. 4a. K562-CD20+ were lysed by all CART constructs except for the single CAR19 constructs, demonstrating target antigen-restricted killing. Similar results were seen with the other leukemia cell lines tested. Single- and tandem-CAR T constructs targeting CD19 lysed Raji, NALM-6, and REH; but not 293 T, Fig. 4b, or K562, Fig. 4a. Notably, the 20A single targeting CAR construct had no specific killing activity against the CD20-negative REH line, but did demonstrate killing of NALM-6, which has low but detectable levels of CD20 surface expression. In addition, the tandem-CAR 1920, which appeared to show lower binding to CD20 peptide than to CD19-Fc by flow cytometry, also has lower cytotoxicity against K562-19^+^ and K562-20^+^, but not against the CD19^+^ CD20^−^ REH. This may suggest that the 1920 tandem-CAR is inferior to 2019 tandem-CAR for some tumor targets.

**Fig. 5:**
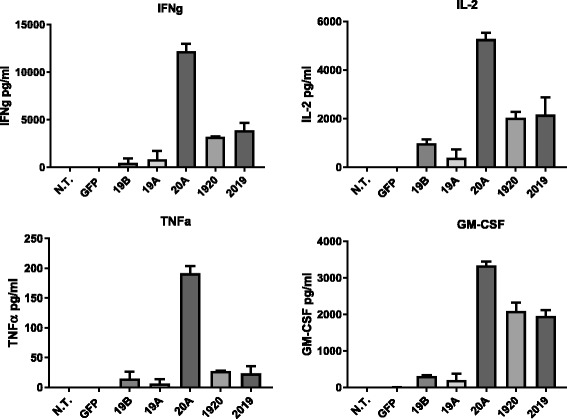
CAR T cytokine release in response to leukemia cell lines. Cytokine production by CAR-T, listed on the x-axis, upon overnight co-culture with the Raji leukemia line at an E:T ratio of 10:1, was measured using a flow-based bead array. Bars represent mean + SD of replicate samples. Data are representative of three independent experiments performed with CAR T cells from three separate donors

We then examined cytokine secretion by tumor-activated CAR T cells. Co-culture media supernatants were harvested following overnight incubation of CAR-T effectors and the Raji cell line at ratio of 10:1, and analyzed by MACSPlex cytokine-specific bead array which allows simultaneous detection of 12 different cytokines, Fig. 5. All CAR T constructs yielded increases in cytokine levels for IFNγ, TNFα, IL-2 and GM-CSF when co-cultured with Raji cells, in comparison to non-transduced T cells, whereas cytokine levels in the negative control groups N.T. and GFP were undetectable. Notably, the 20A CAR was consistently the highest producer of IL-2, IFNγ, TNFα, and GM-CSF. This was not due to a preferential expansion of the CD4^+^ T cell population, as the small changes seen, either up or down, in the CD4/CD8 ratio were not consistent, Additional file 1: Table S1. Both tandem constructs, CAR 1920 and CAR 2019, yielded strong induction of cytokines in the presence of tumor target that was similar in magnitude, and significant *vs* non-transduced controls. Cytokines IFN-α, IL-4, IL-5, IL-6, IL-9, IL-10, IL-12p70, IL-17A, also probed in MACSPlex cytokine array were not detected at significant levels in our samples.

**Fig. 6:**
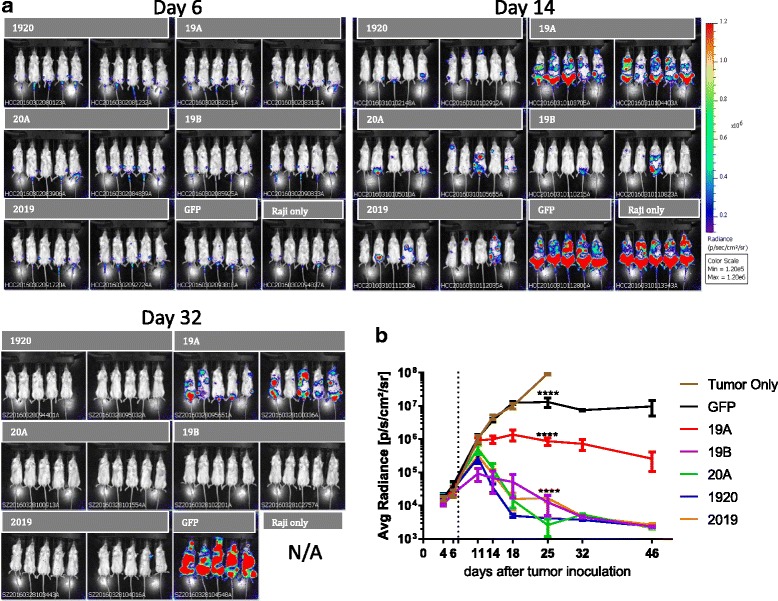
In vivo activity of CAR T constructs. NSG mice were injected *i.v.* with Raji-luciferase cells on Day 0, and treated with CAR T cells on day 7. **a** Bioluminescent images of the tumor burden in mice treated with singe and tandem-CAR T constructs on day 6, 14, or 32, post tumor engraftment are shown. Above each group is a listed the CAR-T used for treatment. **b** Time course of tumor growth based on mouse whole body bioluminescence. 10 mice per CAR T treatment group, and five mice per control group were studied. Mean signal per mouse ± SD is plotted. Statistical analysis for Day 25 (the last time point when subjects in the no treatment control group remained alive) is shown, using two-way ANOVA followed by Dunnett’s multiple comparisons test vs no treatment group. Mean + SD, ***P < 0.001

To evaluate the in vivo activity of CAR-modified human T cells, 0.5 × 10^6^ Raji-Luc cells were injected *i.v.* into NSG mice on day 0. The presence of engrafted leukemia was verified by imaging on day 6 and mice randomized into experimental groups. On day 7, 10 × 10^6^ human T cells transduced with CAR constructs were injected *i.v.* and disease monitored by IVIS imaging. Representative images show the progression or regression of disease in each group, Fig. 6. In animals dosed with 20A single CAR or tandem-CARs 1920 or 2019, tumor burden peaked on day 11, and by day 18 subsided to below pre-treatment level. Tumor elimination in single CAR19 groups was slower by comparison. Interestingly, CAR 19B, in which ScFv heavy and light chains were connected by a Gly-Ser linker, performed better than CAR 19A with a Whitlow linker, Fig. 6b. Overall, both CAR 1920 and CAR 2019 tandem-CAR constructs demonstrated in vivo activity superior of that of single CAR 19 in this model system. Moreover, this data highlights that cytotoxicity, cytokine production, and in vivo activity in an NSG mouse system are each important, but the data must be interpreted together in order to understand the biology of a transduced T cell population.

**Fig. 7:**
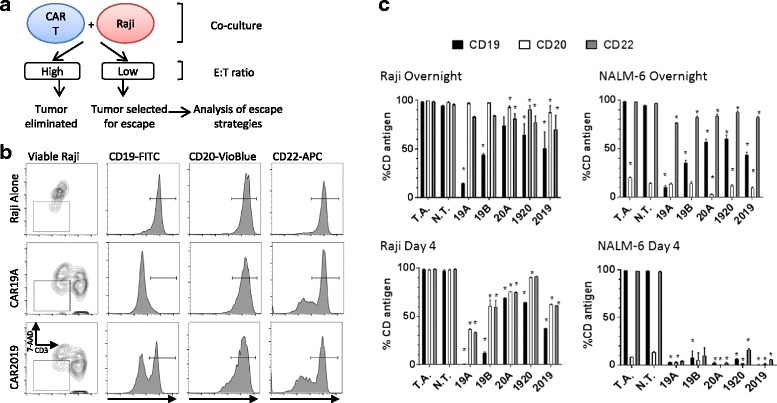
Single CAR19 construct strongly selects Raji tumor escape variants. **a** Diagram of the experimental design for tumor escape experiments. Raji and CAR T cells were co-cultured at E:T ratio of 1:1 either overnight or for 4 days. After overnight incubation and on day 4, cultures were harvested and viable Raji cells examined for CD19, CD20, and CD22 surface expression by flow cytometry. **b** Gating strategy for flow cytometric analysis used to analyze viable Raji cells (7AAD- and CD3-) from co-cultures is shown for representative treatment groups is shown in column 1. Columns 2, 3, and 4 show CD19, CD20, and CD22 expression levels, respectively, when Raji cells were co-cultured with no T cells (row 1), 19A CAR (row 2), or 2019 CAR (row 3). **c** Graphs of CD19, CD20 and CD22 surface expression (*solid*, *open*, *gray*, respectively) in surviving Raji and NALM-6 cells after overnight or 4 days of co-culture with CAR T cells, as listed on x-axis, as determined by flow cytometry. Bars represent group means + SD. Statistical analysis was performed by one-way ANOVA followed by Dunnett’s multiple comparisons test vs N.T. (non-transduced T cells) control from the same donor, *p < 0.05. T.A.- tumor alone control group

Having established a set of mono- and bi-specific CARs, we wanted to explore their activity further. To explore the comparative efficacy of the tandem-CAR constructs in vitro, especially in light of recent descriptions of leukemia escape mechanisms, we used the Raji cell line (which expresses CD19, CD20, and CD22) and the NALM-6 cell line (which expresses CD19, CD22, and low levels of CD20) to explore the ability of leukemia cell lines to escape the strong effector activity of CAR-T, Fig. 7a. To model leukemia cell escape under CAR T immune pressure, CAR-T were co-incubated with leukemia cells at a low effector to target (E:T) ratio of 1:1. When the E:T was higher, all leukemia cells were eliminated. After short-term (overnight) or longer-term (4-day) co-culture, the expression of three B cell markers on the leukemia cell lines’ surface were analyzed by flow cytometry (Fig. 7b, c). CD22 was of interest because it is not specifically targeted by our CAR-T cells and its loss may indicate the onset of a more general immune escape program, generating multiple antigen-loss variants.

When Raji was co-cultured with CAR19-T cells overnight, the CD19 antigen was rapidly down-modulated from the cell surface, Fig. 7b, c. CD20 was relatively constant, and in CD22 a minor population of antigen negative cells began to appear. When the tandem construct 2019 was co-cultured with Raji, CD19 was down-modulated, CD20 was also moderately down-modulated, and close to half of CD22 expression was also lost. When examined at day 4, all B cell antigens were greatly reduced, Fig. 7c. Targeting CD19 alone (constructs 19A and 19B) had the highest impact on CD19 expression. The tandem construct 2019, had a greater impact than the alternate 1920 construct, indicating that stronger immune pressure was mediated by 2019. Whether in short term or long-term co-culture, CD20 is less able to be lost from cells surviving immune pressure. In comparison to the single CD20-targeting construct 20A, the tandem construct 2019 generated more CD19 epitope loss, but appeared to exert similar combined pressure on the other targeted antigen, CD20, and the non-targeted antigen CD22.

When the NALM-6 leukemia cell line was used as a target, little impact was seen on CD20 expression except when the CD20-targeted CAR 20A was utilized (Fig. 7c). This again may reflect a greater resistance to altering CD20 expression on the surface of the leukemia cell line. In overnight co-cultures, CD19 expression was again highly plastic, with antigenic loss on the surviving cell population readily demonstrated. On day 4, few NALM-6 cells were available for study. This is likely due to the far greater sensitivity of NALM-6 to both indirect cytokine and direct cell-mediated killing effects. Notably, no CD22 positive-only cells were detected, indicating overall sensitivity of this line to immune effector mechanisms, i.e. non-selective cell loss.

**Fig. 8:**
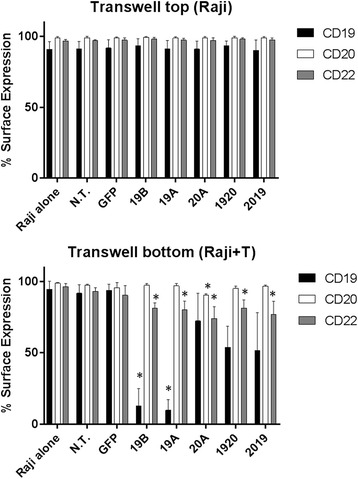
The down-modulation of CD19, CD20 and CD22 on Raji surface requires direct contact with CART cells. Multi-well plates with transwell inserts were used in this experiment. At the bottom of each well, 5 × 10^5^ each of Raji and CAR T cells were combined, and in the transwell upper portion 2.5 × 10^5^ Raji cells were cultured in the absence of T cells. After overnight incubation, cells from the upper transwell compartment and from the bottom compartment were harvested, and viable Raji cells were examined for CD19, CD20, and CD22 surface expression by flow cytometry (*black*, *light grey*, and *dark grey* bars, respectively. Surface expression for each marker with reference to the specific CAR T included in the lower compartment (x- axis) is shown. Bars depict mean + SD of three independent experiments performed using CAR T cells originating from three different donors. One way ANOVA, Dunnett’s multiple comparisons test *p < 0.05

Modulation of the expression of tumor surface molecules by pro-inflammatory cytokines in chronic lymphocytic leukemia has been previously described [15]. Since CAR T cells elaborate high levels of pro-inflammatory cytokines when exposed to tumor cells, as shown in Fig. 5, we endeavored to determine whether the down-modulation of CD19, CD22 or CD20 on Raji cells following co-incubation with CART cells is a direct effect of CAR T-tumor cell contact, or is due to soluble factors released to the medium by CAR T cells. Raji and CAR T cells were combined at the bottom of a transwell plate at an effector to target (E:T) ratio of 1:1. In the upper compartment, we placed Raji cells only. Following overnight incubation, cells from the transwell compartment as well as from the bottom of the well were harvested and analyzed by flow cytometry cell surface expression of CD19, CD20 and CD22 on viable Raji cells (Fig. 8). In agreement with our previous results, Raji cells co-incubated with CAR T cells demonstrated a dramatic reduction in CD19 surface expression by CARs 19A and 19B, and a more modest but significant reduction in CD20 and CD22. By contrast, Raji cells recovered from the upper transwell compartment, preserved full expression of CD19, CD20 and CD22 and were indistinguishable from the negative control groups: N.T., GFP and Raji alone. Therefore, the down-modulation of CD19 Raji expression by CAR T cells 19A and 19B is a direct effect of Tumor:CAR T contact.

**Fig. 9:**
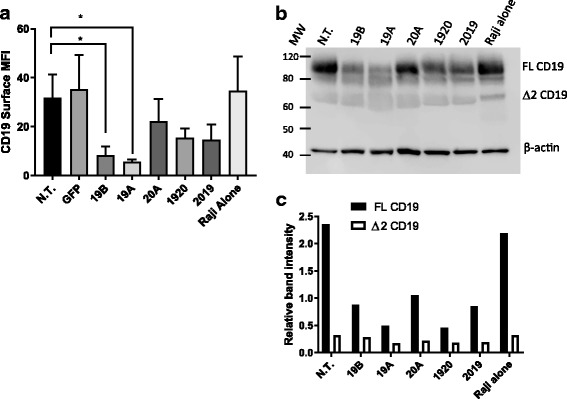
Down-modulation of CD19 full-length protein and CD19 splice variant by CAR19 constructs. Raji cells were co-incubated with CAR T cells at a 1:1 E:T ratio. After overnight incubation, T cells were removed from co-incubated cell populations using magnetic beads. CD19 expression on purified Raji populations was investigated by flow cytometry and Western blot. **a** Raji cell samples were stained with anti-CD19 antibody and acquired by flow cytometry. Median fluorescence intensity for Raji cells representing each treatment group is shown. Bars depict mean + SD of three independent experiments performed using CAR T cells originating from three different donors. One way ANOVA, Dunnett’s multiple comparisons test *p < 0.05. **b** Lysates of purified Raji cells from each of the co-incubated groups (CAR-T identity is listed above each line) were resolved on a 4–12% SDS polyacrylamide gel as described in the Methods, and probed with antibodies targeting the C-terminus of CD19 molecule, or β-actin (loading control). **c** The intensity of specific immunoreactive bands representing full-length CD19 protein (FL CD19), and the exon 2 spliced CD19 variant (Δ2CD19) was quantified using Image Studio software (LI-COR Biosciences). Relative band intensity of CD19 bands was calculated as signal CD19/signal β actin

It has been recently reported that CD19–bearing tumors can evade elimination by CAR19 through preferential expression of a splice variant missing exon 2, which removes a portion of the CD19 extracellular domain that contains the binding epitope for FMC63 [8]. To explore CD19 plasticity on the Raji leukemia cell line, a co-culture experiment was carried out. Raji cells were co-cultured with CAR T overnight, then analyzed by flow cytometry to assay CD19 expression. A significant reduction in CD19 MFI was measured in both 19A and 19B experimental groups (Fig. 9a). Immunomagnetic beads were then use to deplete the co-culture, and the purified Raji cells were analyzed by Western blotting (Fig. 9b, c).

In order to evaluate whether splicing of exon 2 may have contributed to the diminished expression of full length CAR 19 protein, we performed Western blot analysis, as previously described [8]. We found that the Raji cells express both full length and the Δ2 spliced CD19 isoforms in standard culture (Raji alone group), Fig. 9b. The intensity of Western blot bands was analyzed in order to quantify the relationship between the expression of full-length CD19 and its Δ2 splice variant (Fig. 9c). Decreased expression of the full-length CD19 isoform occurred in groups treated with CAR19 constructs 19A, and 19B, in agreement with our flow cytometry results. Conversely, the expression of Δ2 spliced CD19 remained relatively stable regardless of CAR T treatment (Fig. 9c). When CAR T were removed from co-culture and purified Raji incubated alone, all treatment groups re-expressed the target antigens CD19, CD20, and CD22 at non-treated levels (98%-and above, not shown) by day 4. This demonstrates a very dynamic regulation of cell surface protein expression.

**Fig. 10:**
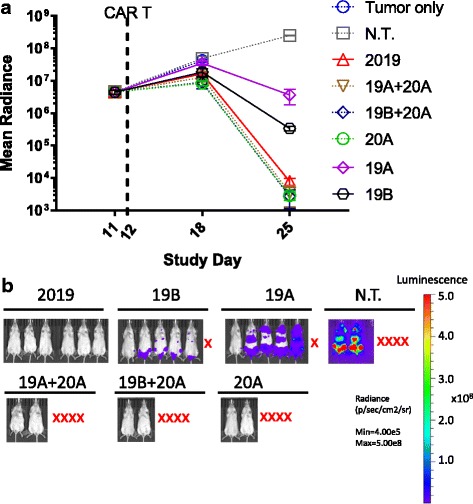
In vivo activity of CAR T cells in a high tumor burden model. NSG mice (n = 6) were injected *i.v.* with Raji-luciferase cells on Day 0, and treated with CAR T cells, as indicated in the figure, on day 12. **a** Disease burden is plotted as the average bioluminescent signal (mean radiance [p/s/cm^2^/sr]) ± SEM. Groups with less than half of the mice surviving to day 25 are plotted as dotted lines. Groups where more than half survived are plotted as solid lines. **b** Bioluminescent images of the tumor burden in mice treated with singe and tandem-CAR T constructs as indicated in the plot above on day 25 post tumor engraftment are shown. *Red X* indicates mice that did not survive to study day 25

To further explore differences in tumor elimination between single and tandem-CARs, an additional study was performed in which Raji tumors were allowed to grow to day 12, as opposed to day 7, prior to CAR T administration, Fig. 10. Day 12 tumor-bearing mice received transduced T cell product with 5 × 10^6^ CAR T^+^ cells per mouse for the single CARs, or the tandem 2019 CAR. We also co-administered two T cell products simultaneously, containing separate transductions of the CD19A + 20A or the CD19B + 20A CARs. A total of 2.5 × 10^6^ CAR T cells were given for each construct (for a total of 5 × 10^6^). We did not pursue dual-transduction strategies as the number of variables was simply too great.

The tandem-CAR2019 achieved strong reduction in tumor burden, in the absence of mortality, whereas the combination treatments 19A + 20A and 19B + 20A were effective but highly toxic, with only two out of six mice surviving to day 25 in each group. For the single CAR 19A and 19B groups, the tumor burden remained relatively high and only five out of six mice in each group survived to day 25. In the single 20A group, the tumors were cleared efficiently, but only two mice out off six survived. Surprisingly, the study highlighted the toxicity of CAR administration, which was greatest in the single 20A group or when 20A was combined with 19A or 19B CAR T cells.”

## Discussion

**Fig. 11:**
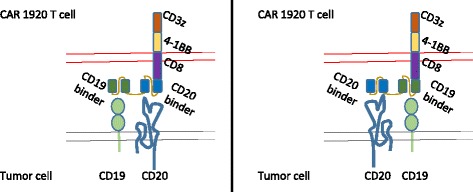
Schematic representation of Tandem-CAR T cells targeting CD19 and CD20 tumor antigens. The tandem-CAR 1920 (*left*) and 2019 (*right*) are comprised of tandem extracellular targeting domains linked in frame to CD8-derived hinge and transmembrane domains, followed by the 4-1BB costimulatory domain, and the CD3 zeta activation domain. Each CAR T construct is capable of activation via binding to either CD19 or CD20 tumor antigens, or both

The linkage of anti-CD19 and anti-Her2 domains into a single tandem-CAR (termed a TanCAR by the authors) reduced to practice what many had proposed in theory, that the generation of a dual-specific chimeric antigen receptor (CAR) was possible [16]. In our drive to create improved CARs for the adoptive immunotherapy of hematologic malignancies, we linked anti-CD19- and anti-CD20-based binding motifs into a single transmembrane glycoprotein to create a set of tandem-CARs. These CAR constructs are capable of activation via binding of either CD19 or CD20 tumor molecules, as depicted in Fig. 11, and are effective both in vitro and in vivo against model leukemia cell lines. Our standard animal model did not reveal a clear advantage or establish a preferred order of the CD19 and CD20 scFv within the CAR structure itself. Nevertheless, the 2019 CAR construct showed better binding of the CD20-peptide staining reagent by flow cytometry, and improved killing of some tumor cells lines in vitro, Figs. 2 and 4. Furthermore, analysis of immune pressure in overnight and 4-day co-culture experiments indicated that the 2019 CAR may exert stronger immune pressure on the target leukemia cell lines, Figs. 7 and 9.

With regard to the polypeptide sequences of our CAR proteins, we note the effectiveness of linking both the V_H_ and V_L_ domains with a poly-glycine linker (GGGGS), as well as the linking of the two independent scFv in the tandem-CAR structures. This is important as the length of the V_H_ and V_L_ linking sequences, as well as their amino acid composition, have been shown to govern diabody formation and proper folding of the scFv domain [11]. Our data also support the findings of Zah, et al., who were able to link the independent CD19 and CD20 scFv domains using the (GGGGS) sequence [17]. As demonstrated by Western blot analysis, the intensity of the native CD3-zeta chain bands indicates that the transcription or translation of TCR-associated transcripts like the zeta chain are not overwhelmed or displaced by CAR-transcription using an EF-1-alpha driven CAR payload in a LV system, Fig. 3.

Demonstration of the ability to bind target protein by the scFv encoded by the CAR and tandem-CAR vectors was achieved using a set of unique tools generated by the expression of recombinant fragments of CD19 and CD20, Fig. 2. Previous reports have used Protein L, which binds kappa light chain sequences, to stain for cell surface CAR expression [13]. For both FMC63-based scFv CARs specific for CD19 (constructs 19A, 19B), protein L bound well, although the original Whitlow linker had a brighter MFI. This held true for staining with the recombinant CD19 fragment as well, but the difference was less pronounced. Protein L and recombinant CD20 peptide gave very similar results for the Leu16-based anti-CD20 CAR (construct 20A). In the tandem-CARs, Protein L staining was equivalently strong. The anti-CD19 scFv binding of target peptide was essentially equivalent in tandem and single CARs, but signal for CD20-biotin-based staining was diminished in construct 1920, in comparison to 2019 (Fig. 2b), which may indicate steric hindrance for binding to CD20 in this construct. In the CD20 negative leukemia cell lines, we saw a somewhat lower level of lysis with the CAR 1920 as opposed to the CAR 2019 tandem vector. One possible explanation for this functional disparity is that in tandem-CARs the CD19 binder has to be placed close to T cell membrane to match the distance of the CD19 epitope from the tumor cell surface. This notion is supported by the example of ROR1 CARs, where the length of extracellular spacer was decisive in determining the CAR tumor recognition [18]. Interestingly, CD19 binder placement proximal to T cell membrane was also required for optimal function of another tandem-CAR currently in development, the CD22_CD19 CAR (W. Haso, unpublished observations). Better antigen binding by the 2019 CAR may reflect that the CD20 binder requires the carboxy terminus to be untethered for the proper V_H_ to V_L_ folding or that in the 1920 construct the CD20 binding site is obscured by the adjacent linker-CD19 domains.

In vitro cytokine release activity also demonstrated tandem-CAR activity, Fig. 5. However, the CD20-specifc CAR 20A was superior to tandem and single CD19 CAR constructs in the production of Th-1 like cytokines: IFN-gamma, IL-2, and TNF-alpha, and GM-CSF. Both tandem-CARs were similar in cytokine production and were greater than the single 19A and 19B constructs. The level of cytokine produced by the tandem-CAR constructs 1920 and 2019 may be primarily driven by CD20 recognition, and reflect another tandem-CAR advantage, namely that optimal features from each binder were preserved for better potential therapeutic effect.

In vivo CAR 19B performed better than CAR 19A, Fig. 6. This may be due in part to the superior ability of 19B to produce IL-2 and thus sustain T cell activity in the NSG mouse model, which we did not supplement with the addition of human cytokines. Taken together these data indicate that cytokine production is important in predicting the in vivo activity of anti-leukemia CARs in the NSG mouse system.

We then tested the ability of CAR-mediated immune pressure to modulate leukemia phenotypes. To this end, we found that although effector to target ratios of 5:1 and above effectively eliminated leukemia cells in CTL assays, when the lower effector to target ratio of 1:1 was used, we could still harvest the surviving leukemia cells and analyze their surface antigen expression. Flow cytometry allowed us to gate for viable cells, exclude T cells, and quantify antigen expression, Fig. 7a, b. After overnight incubation with the 19A and 19B CARs, strong down-modulation of CD19 was seen. The tandem-CARs induced less antigenic loss, but there were far fewer cell remaining. CD20 expression was modulated less. Interestingly, some CD22- down-modualtion was also detected. This may indicate that both antigen-specific and non-antigen-specific mechanisms of escape are possible in the Raji cell population analyzed. We then carried out both overnight and 4-day CAR co-incubations with the Raji and NALM-6 cell lines, Fig. 7c. The analysis of NALM-6 was limited by the more general sensitivity of NALM-6 to activated lymphocytes. Even at the low 1:1 E:T ratio, few NALM-6 leukemia cells from the co-cultures were available for analysis on day 4. After overnight incubation, strong down-modulation of CD19 expression was seen, with CAR 19A and 19B LV-transduced T cells having the greatest effect. The tandem-CARs and surprisingly the 20A CD20 CAR all had a measurable effect on CD19, (Fig. 7c). Also of note, CD22 expression decreased by 15–20%, indicating that non-targeted B cell differentiation antigens are impacted as well.. Using the Raji cell line, we were better able to observe the effects of CAR-mediated immune pressure on CD20. In CAR-T co-culture experiments, CD20 was less variable with respect to loss of surface antigen expression than the non-targeted antigen CD22. As with NALM-6, CD19 down-modulation was rapid and occurred in the vast majority of cells. After 4 days of immune pressure in Raji cells, the tandem-CARs 2019 and 1920 appeared to maintain immune pressure as all three B cell antigens were moderately down-modulated. CD19 expression remained strongly down-modulated, especially with the CD19 single specificity vectors. The 20A CAR immune pressure, both overnight and at day 4, showed a lesser ability to select for escape mutants, indicating perhaps a lesser ability for Raji cells to dispense with or alter its expression.

Down-modulation of tumor antigen surface expression by soluble cytokines produced by inflammatory cells has been documented in chronic lymphocytic leukemia [15]. We investigated whether the soluble factors present in the medium of co-cultured CAR T and tumor cells are responsible for the down modulation of CD19, CD20 and CD22 on Raji cells in a transwell co-culture assay. We found that direct contact between Raji tumor cells and CAR T cells was necessary to down-modulate Raji surface expression of CD19, CD20 and CD22.

In order to probe the mechanism of CD19 loss with regard to antibody staining by flow cytometry, we used the findings of the Thomas-Tikhonenko lab to investigate the prevalence of CD19 isoforms in Raji cells exposed to CAR T [8]. To that end we co-cultured Raji cells with CAR-T, then analyzed them by flow cytometry at the end of overnight incubation to demonstrate reduction in CD19 expression. Subsequently, we used immunomagnetic beads to deplete the co-culture of T cells. The separated Raji cells were analyzed by Western blotting. Surprisingly, we found that the reduction in the full-length CD19 isoform was mainly responsible for the observed decrease in CD19 staining, and the levels of Δ2 CD19 isoform remained relatively stable regardless of treatment.

Given that the variation in B cell surface antigen expression was so profound, we wanted to investigate its relative permanence. In other words, was this a true mutational effect, or was it a phenotypic accommodation? To address this question, the CAR T-separated Raji cells were returned to culture, and then analyzed by flow cytometry again on day 4. All tumor populations demonstrated full recovery, with CD19, CD20, and CD22 expression being greater than 98%.

Tumor antigen escape is one of the major challenges facing the field of adoptive cell therapy today. Despite significant progress achieved in the treatment of relapsed or refractory ALL using CAR19, tumor escape by downregulating the CD19 epitope on tumor cells has been reported [5, 19, 20], and is responsible for a significant portion of disease relapse. It has become clear that combinatorial approaches will be necessary to overcome tumor escape in hematologic malignancies, especially in disease types that are more challenging to treat than pediatric pre-B-ALL, such as NHL, reviewed in [21]. Furthermore, as evidenced for CAR19 treatment of leukemia and as was also shown in an in vivo model of PSCA and MUC1-positive tumors [22], heterogeneous target antigen expression may lead to tumor escape variants when a single CAR T therapy is used. Therefore, targeting multiple tumor antigens with a single CAR T therapeutic product may mitigate tumor antigen escape.

Sotillo and co-workers [8], demonstrated that CD19 loss occurs in primary disease as well as leukemia and lymphoma cell lines under CAR19 pressure by a variety of mechanisms, including both mutations and expansion of CD19-negative variants, as well as alternative splicing that yields the exon 2-deficient CD19 variant. Recent studies have shown that after prolonged CAR-T immune pressure on CD19 antigens, tumors may also escape detection by reverting to a CD19-negative myeloid phenotype [23, 24]. In our experimental model, CD19 downregulation on Raji leukemia in the presence of CD19 CAR constructs 19A and 19B was rapid, with a significant loss of detected CD19 expression occurring after just an overnight co-culture with CAR T. The loss of CD19 expression was restored completely within 4 days after the CAR T cells were removed from culture. Given the rapidity of the change we are measuring, and considering that Raji cells replicate approximately once every 20 h, CD19 downregulation by an actual CD19 loss mutation and the preferential expansion of CD19-negative lymphoma clones in our model is unlikely. Furthermore, we have determined that in the presence of the single CAR19 constructs 19A and 19B, the amount of full length CD19 protein in Raji whole cell lysates is reduced, in concordance with the diminished CD19 staining as probed by flow cytometry, whereas the amount of exon 2 spliced CD19 remains relatively stable regardless of the type of CAR T used. Therefore, the alternative splicing of CD19 exon 2 is not modified by CAR-mediated downregulation of full-length CD19.

CD19 is a B cell co-receptor, and it acts a positive regulator of maturation, proliferation and survival in early and late B cell development [25, 26]. CD19 is internalized together with the B cell receptor following its engagement by ligand. Similarly, CD19 can be internalized following binding by specific antibody, a fact that is being exploited in antibody-conjugate (ADC) therapy. Internalization of CD20 from the surface of B cells is also known to occur following the use of the therapeutic antibody rituximab [27]. One could speculate that CAR19 pressure would cause CD19 internalization as well. However, in our system the mere internalization of CD19 could not explain the fact that the level of full-length CD19 protein is diminished following 19A and 19B treatment, as shown by Western blot. Thus, CD19 protein downregulation must occur at the transcriptional or translational level, or by increased degradation of the CD19 protein upon CAR-driven internalization. This effect is very dynamic, and is reversed soon after the CAR19 pressure in removed.

The significance of the reduction in CD19 expression by exposure to CAR T therapy for tumor growth remains to be fully elucidated. Mechanistically speaking, CD19 reduces B cell activation thresholds by promoting B cell receptor-antigen micro cluster formation, and initiating several downstream signaling pathways [28]. CD19 is typically thought of as a promoter of lymphomagenesis, and has been shown to drive B cell proliferation via a positive feedback loop with MYC oncoprotein [29, 30]. However, CD19 is downregulated on some types of B cell leukemias, including CLL, B-PLL, SLVL, and MCL [9]. It is therefore possible that in some instances, such as when selective pressure is applied by CD19-targeted CART cells, CD19 loss may confer a tumor growth advantage. The plasticity of CD19 evidenced in leukemic cells may be a characteristic preserved from normal B cell biology, now providing an additional survival advantage to the malignancy.

Finally, we tested the activity of tandem-CARs versus single CARs in a more advanced disease setting, with a higher tumor burden. We also compared a simplified approach where two CAR products, each expressing a single CD19- or CD20-CAR were combined into one effector population, Fig. 10. The CD2019 CAR was the only treatment group that was able to control the tumor in which all the subjects survived to study day 25. The single CARs 19A and 19B on their own did not have as strong an anti-disease effect and one mouse was lost from each group. The 20A CAR on its own or in combination with either CD19 CAR did apparently clear mice of disease, but four of the six mice did not survive to day 25. This may be due to the greater probably of the tandem 2019 CAR to be activated by the tumor cell, and its more moderate cytokine production profile as compared to the 20A CAR, Fig. 5. In this study we could not differentiate between deaths due to advanced disease or CAR related toxicity. However, we suspect that 20A CAR-related toxicity may have played a role, because of the complete clearance of disease from the surviving mice. Although alloreactivity may play a role in all of our studies, since we are using model tumor cell lines, the lack of activity of GFP or N.T. controls in any of our assays, demonstrates that it is not a major effect.

## Conclusions

Our studies on their own do not prove that CAR T immune pressure generates durable escape mutants, but clinical experience to date has shown that CD19 negative relapse is not a rare event [8, 19, 20]. Moreover, we demonstrate that CD19 antigen modulation is a very rapid event, and that the targeting two antigens at the same time may be a reasonable approach to address this issue. The high tumor burden study highlights an interesting new biology that could be attributed to the tandem-CAR T product and may indicate a superior CAR design format for translational studies. Alternatively, we have struck a unique balance between effective in vitro activity, notably cytokine production and killing activity, that needs to be ascertained for optimal in vivo activity by considering both the CAR expressed by the effector cell and the overall disease burden.

## Additional file

Additional file 1: Table S1. CD4/CD8 ratio of CART -transduced cell preparations. **Figure S1.** CAR amino acid sequences. (PDF 5590 kb)

## Abbreviations

7AAD: Seven-actinomycin D
AA: Amino acids
AF: Alexa fluor
ALL: Acute lymphoblastic leukemia
ANOVA: Analysis of variance
APC: Allophycocyanin
CAR T: Chimeric antigen receptor T cells
CD: Cluster of differentiation
CLL: Chronic lymphocytic leukemia
CPS: Counts per second
CTL: Cytotoxic T lymphocyte
ELISA: Enzyme linked immunosorbent assay
FBS: Fetal bovine serum
Fc: Fragment crystallizable region
FITC: Fluorescein isothiocyanate
GFP: Green fluorescent protein
GM-CSF: Granulocyte macrophage colony stimulating factor
GMP: Good manufacturing practice
HCL: Hairy cell leukemia
HEK: Human embryonic kidney cells
Her2: Human epidermal growth factor receptor two
IFNγ: Interferon gamma
IL-2: Interleukin two
IU: International units
Luc: Firefly luciferase
LV: Lentiviral vector
MCL: Mantle cell lymphoma
MOPS: Three-(N-morpholino)propanesulfonic acid
N.T.: Non-transduced T cells control from same donor
NSG: NOD.Cg-*Prkdc* ^*scid*^ *Il2rg* ^*tm1Wjl*^/SzJ
OBI: Oklahoma Blood Institute
PE: Phycoerythrin
PLL: Prolymphocytic leukemia
ROR1: Receptor tyrosine kinase-like orphan receptor 1
RPM: Revolutions per minute
scFv: Single chain variable fragment
SDS-PAGE: Sodium dodecyl sulfate polyacrylamide gel electrophoresis
SLVL: Splenic lymphoma with villous lymphocytes
T.A.: Tumor alone control group
TNFα: Tumor necrosis factor alpha
V_H_: Variable heavy chain domain
V_L_: Variable light chain domain
