# Supplementary material for: A tandem CD19/CD20 CAR lentiviral vector drives on-target and off-target antigen modulation in leukemia cell lines
Source: J Immunother Cancer. 2017 May 16;5:42. doi: 10.1186/s40425-017-0246-1 (PMC5433150; doi:10.1186/s40425-017-0246-1)
Supplement: Additional file 1: Table S1. — CD4/CD8 ratio of CART -transduced cell preparations. Figure S1. CAR amino acid sequences. (PDF 5590 kb) [file 40425_2017_246_MOESM1_ESM.pdf]

Supplementary files

Supplementary Table 1: CD4/CD8 ratio of CART -transduced cell preparations.

	**Donor 1**	**Donor 2**	**Donor 3**
CAR T	CD4/CD8	CD4/CD8	CD4/CD8
N.T.	4.4	7.4	3.2
GFP	2.6	6.3	2.4
19A	4.6	11.4	2.5
20A	3.2	12.4	2.9
19B	5.9	13.3	2.4
1920	5.2	6.2	2.2
2019	4.7	8.1	2.1

Legend for Supplementary Table 1

The CD4/CD8 ratio in CAR T cell preparations from three separate donors was determined by flow cytometry. The $CD4^+$ and $CD8^+$ T cells were co-purified from buffy coats, cells were activated with TransAct CD3 CD28 reagent in the presence of IL-2, transduced with LV as described in Materials and Methods, and assayed for CD4/CD8 composition on culture day 10, when immune function was assayed. The ratio of CD4/CD8 cells for each of the three donors is noted in columns, and CAR T groups noted in rows.

Supplementary Figure 1. CAR-encoding vectors.

(a) pCD19CD20 CAR Vector Configuration:

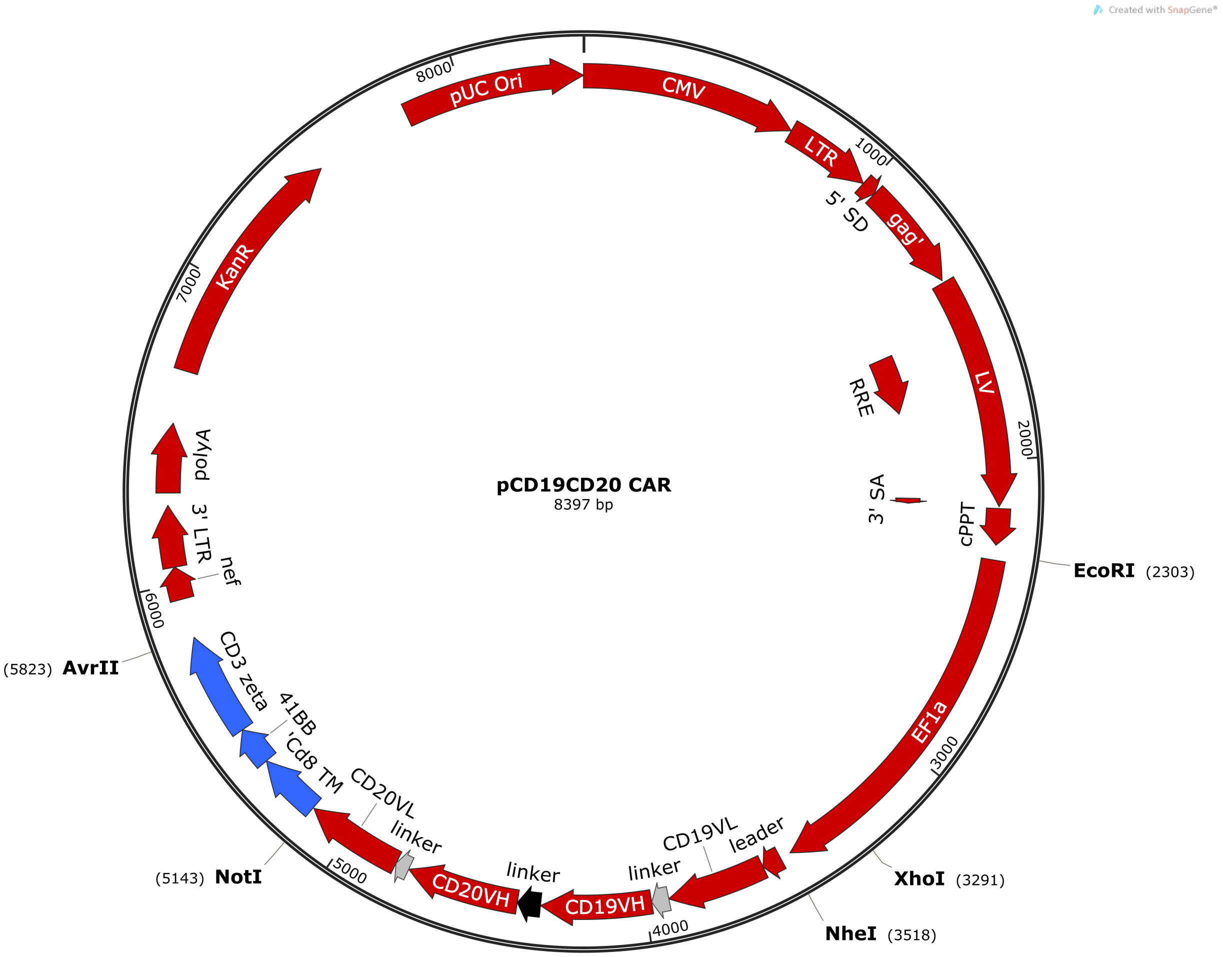

(b) pCD20CD19 CAR Vector Configuration:

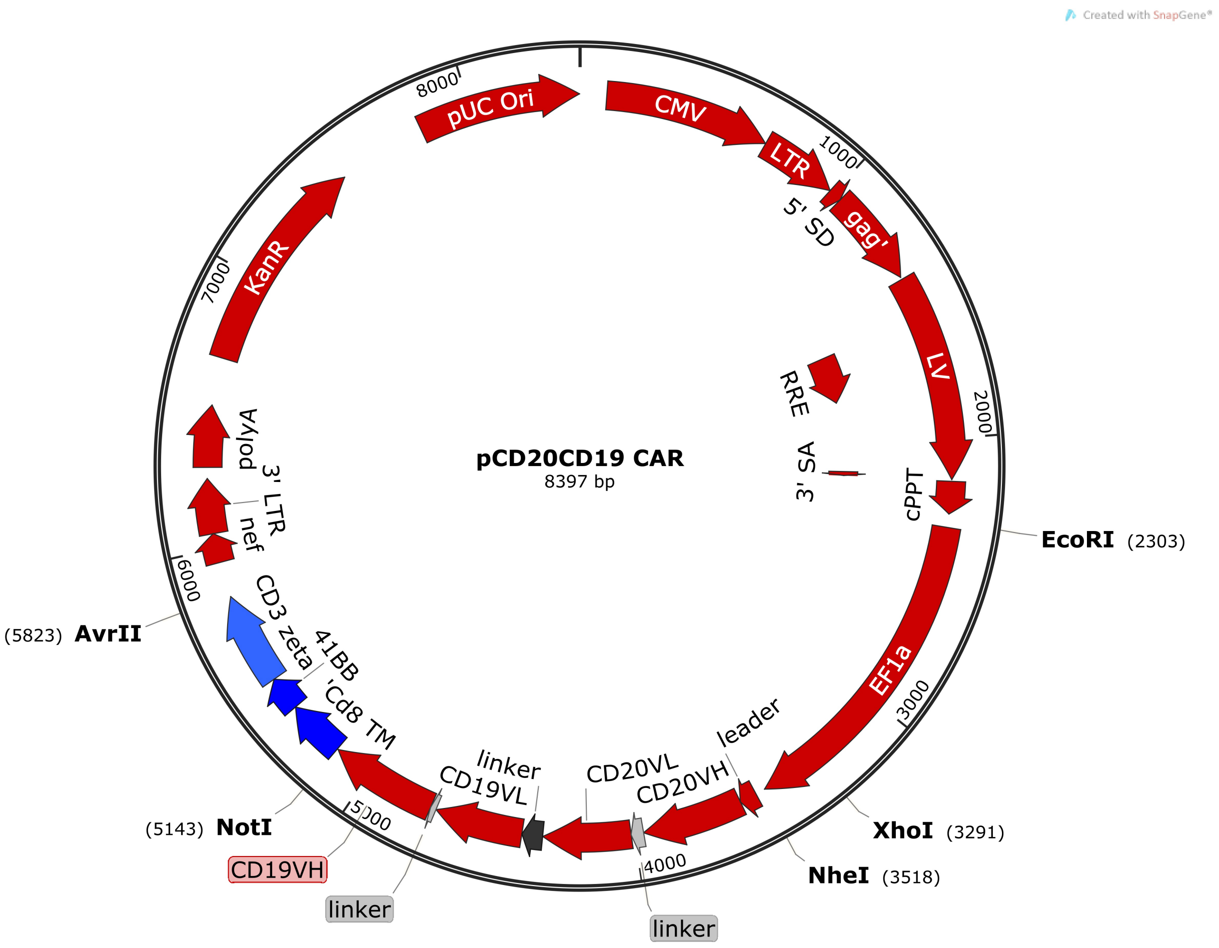

Legend for Supplementary Figure 1:

Tandem CAR lentiviral vectors (LV) encoding the (a) CD19_20 and the (b) CD20_19 CARs were expressed using lentiviral backbone plasmids featuring a human EF-1alpha internal promoter (EF1a), leader sequence (leader), VH and VL sequence from FMC63 and Leu 16 antibodies (CD19VL, CD19VH, CD20 VL, CD20VH, respectively), inter-chain linker sequence (in grey), and intra-scFv sequence (in black), linked to CD8, 41BB, and CD3zeta signaling domains (in blue).
